# An Improved Orthogonal Matching Pursuit Algorithm for CS-Based Channel Estimation

**DOI:** 10.3390/s23239509

**Published:** 2023-11-29

**Authors:** Lu Si, Weizhang Xu, Xinle Yu, Hang Yin

**Affiliations:** 1 State Key Laboratory of Media Convergence & Communication, Communication University of China, Beijing 100024, China; 2 Engineering Research Center of Digital Audio & Video, Ministry of Education, Communication University of China, Beijing 100024, China

**Keywords:** compressed sensing, single-carrier frequency domain equalization, orthogonal matching pursuit, simultaneous orthogonal matching pursuit, Cramér–Rao lower bound

## Abstract

Wireless broadband transmission channels usually have time-domain-sparse properties, and the reconstruction of these channels using a greedy search-based orthogonal matching pursuit (OMP) algorithm can effectively improve channel estimation performance while decreasing the length of the reference signal. In this research, the improved OMP and SOMP algorithms for compressed-sensing (CS)-based channel estimation are proposed for single-carrier frequency domain equalization (SC-FDE) systems, which, in comparison with conventional algorithms, calculate the path gain after obtaining the path delay and updating the observation matrices. The reliability of the communication system is further enhanced because the channel path gain is calculated using longer observation vectors, which lowers the Cramér–Rao lower bound (CRLB) and results in better channel estimation performance. The developed method can also be applied to time-domain-synchronous OFDM (TDS-OFDM) systems, and it is applicable to the improvement of other matching pursuit algorithms.

## 1. Introduction

Channel estimation is among the key technologies in modern wireless communication systems, and data-assisted channel estimation offers remarkable advantages, such as low errors and low complexity, and it is extensively applied to modern wireless transmission systems. Conventional channel estimation methods do not use priori information while transmission channels have sparse properties but focus only on the maximum delay spread of the channel, so they lead to the overuse of bandwidth and energy due to the large overhead of the reference signal. Compressed-sensing (CS)-based methods take advantage of the fact that the propagation multipath of wireless broadband channels usually has time-domain sparsity characteristics [1] and adopt a parametric estimation method to complete channel state detection. By estimating the path delay, path gain, and phase parameters of each propagation path, the parametric estimation method is able to achieve superior channel estimation performance with fewer reference signals, and thus, it improves the effectiveness and reliability of the system. Currently, CS-based sparse channel estimation is among research hotspots.

Sparse signal reconstruction is the core of CS theory [2,3]. References [4,5,6] provided reviews of the CS technique and also a comprehensive overview of various applications of sparse representation in wireless communications. Reference [4] focused on the sparse recovery algorithms and discussed some comparisons among them, and it also presented several applications of compressed sensing, such as images and videos, compressed transmission data, systems communication, and detection and recognition systems. Reference [5] identified the potential applications and research challenges of sparse representation in fifth-generation (5G) and Internet-of-Things (IoT) networks. Reference [6] listed some future research directions for CS techniques in wireless applications. Reference [7] investigated the application of a CS technique and an OMP algorithm to reconfigurable-intelligent-surface (RIS)-assisted wireless communication systems.

There are two main types of methods for signal reconstruction algorithms for CS: convex optimization methods and greedy algorithms. Because of the unacceptable computation time and complexity of convex optimization algorithms, greedy algorithms are more commonly applied. A greedy algorithm is not an optimization algorithm, and unlike convex optimization algorithms, it does not have an objective function and does not minimize it, but it finds the positions and coefficients of the non-zero elements of the sparse signal through several iterations to achieve sparse signal recovery [8]. As an important greedy algorithm, the orthogonal matching pursuit (OMP) algorithm is more commonly applied to CS-based channel estimation [9]. Reference [10] used the OMP algorithm to estimate sparse channels with more accuracy than conventional algorithms. Reference [11] provided a detailed description and summary of different CS-based channel estimation methods.

The OMP algorithm can cause reconstructed results to fall into a local optimum. In order to improve estimation accuracy, reference [12] proposed the A*OMP pseudo-greedy algorithm, which utilized the backtracking feature of the A* algorithm. Compared with the OMP algorithm, it had higher accuracy and reconstruction probability. Reference [13] developed an improved A*OMP algorithm and verified its performance in orthogonal frequency division multiplexing (OFDM) systems. The obtained results showed that the proposed method effectively improved the accuracy of channel estimation. The improved algorithm optimized path initialization; meanwhile, in order to avoid unknown errors caused by too many iterations, the difference between the residuals of two adjacent iterations was applied as a condition for the termination of iteration, which resulted in higher estimation accuracy.

Due to an increasing data rate and system bandwidth, the channel responses experienced with adjacent symbols were no longer independent from each other, but they presented a time-dependent joint sparse structure [14]. To describe this feature, a joint sparse model (JSM) [15] was proposed, and accordingly, the simultaneous orthogonal matching pursuit (SOMP) [16,17], which was based on distributed compressed sensing (DCS), has been widely studied. Reference [18] improved the SOMP algorithm by first jointly estimating the same support set and the elements of the channel response for multiple consecutive symbols and then estimating different parts of the support set, symbol by symbol. The obtained results showed that the improved method was applicable to both the JSM-1 and JSM-2 models.

The sparse reconstruction algorithms mentioned above were mostly applied to CS-based channel estimation for OFDM systems employing frequency domain pilots. References [19,20,21] discussed the application of CS-based sparse channel estimation in time-domain-synchronous OFDM (TDS-OFDM) systems, which utilized a known training sequence (TS) in the time domain as a guard interval for data blocks. The frame structure of the single-carrier frequency domain equalization (SC-FDE) system is very similar to that of TDS-OFDM. Reference [22] investigated the application of the OMP algorithm to sparse channel estimation in SC-FDE systems. The algorithm proposed in reference [20] required a small amount of frequency domain pilots, and reference [22] proposed inserting the cyclic prefix (CP) of the TS in the header of a frame. All these methods required additional pilots, which sacrificed spectral efficiency while obtaining superior performance.

For SC-FDE systems, an improved scheme is proposed in this research, which was applied to both the OMP and SOMP algorithms, and the improved algorithms are abbreviated as P-OMP and P-SOMP. The contribution of this research is to focus on the improvement of the algorithmic structure to achieve superior channel estimation performance without resorting to additional pilots or TS. Compared with conventional algorithms, the improved algorithms calculate the path gain after obtaining the exact path delay and updating the observation matrix. With a longer observation vector for estimating the path gain, the improved algorithms reduce the Cramér–Rao lower bound (CRLB) and achieve better estimation performance; therefore, they effectively improve the reliability of the communication system.

Due to the similarity of the transmission frame structure, the proposed method is also applicable to TDS-OFDM systems. In addition, the main processes of all the matching pursuit algorithms are the same, and the proposed method is also applicable to other matching pursuit algorithms. Therefore, the developed method is important for the reliable transmission of wireless communication systems based on the transmission block structure employing time-domain pilots.

Notation: ${\left(\cdot \right)}^{T},{\left(\cdot \right)}^{H},{\left(\cdot \right)}^{†},{\left(\cdot \right)}^{-1}$ and ${\left\|\cdot \right\|}_{p}$ denote transpose, conjugate transpose, Moore–Penrose matrix inversion, matrix inversion, and *l_p_* norm operations, respectively. $I_{n}$ denotes the identity matrix of dimension n.

## 2. Materials and Methods

### 2.1. Wireless Communication System Model Based on CS Channel Estimation

To eliminate the effects of channel multipath and Doppler on the transmitted signal via channel equalization techniques, an accurate channel state has to be obtained. Block transmission systems based on frequency domain equalization, such as OFDM and SC-FDE, have been extensively applied due to their good compromise between system complexity and performance.

#### 2.1.1. OFDM System Based on CS Channel Estimation

Assuming that each OFDM symbol has *N* subcarriers, X(k) denotes the *k*th subcarrier of the transmitted symbol. After channel transmission, the *k*th subcarrier received at the receiver is:

(1) Y(k)=X(k)H(k)+W(k)

In Equation (1), W(k) denotes the noise frequency response of the *k*th carrier, H(k) denotes the channel frequency response of the *k*th subcarrier, and the output of the *N* subcarriers can be expressed as follows:

(2) $$\begin{array}{c} Y\left(1\right)=X\left(1\right)H\left(1\right)+W\left(1\right)\\ \cdots \cdots \\ Y\left(N\right)=X\left(N\right)H\left(N\right)+W\left(N\right) \end{array}$$

In matrix form, it is stated as follows:

(3) $$\left[\begin{array}{c} Y\left(1\right)\\ \vdots \\ Y\left(N\right) \end{array}\right]=\left[\begin{array}{ccc} X\left(1\right) & \cdots & 0\\ \vdots & \ddots & \vdots \\ 0 & \cdots & X\left(N\right) \end{array}\right]\left[\begin{array}{c} H\left(1\right)\\ \vdots \\ H\left(N\right) \end{array}\right]+\left[\begin{array}{c} W\left(1\right)\\ \vdots \\ W\left(N\right) \end{array}\right]$$

that is,

(4) Y=XH+W

where X=diag[X(1),X(2)⋯X(N)] is the diagonal matrix of N×N consisting of transmitted signals, *H* is the frequency domain response of the channel at N×1, *W* is the Gaussian white noise vector in the frequency domain at N×1, and *Y* is the received signal vector at N×1. The relationship between the frequency domain channel response *H* and time domain channel impulse response (CIR) *h* is expressed as follows:

(5) H=Fh

where *F* is the Fourier transform matrix of N×N. Substituting Equation (5) into Equation (4) yielded the following:

(6) Y=XFh+W

CIR *h* is a vector of N×1, in which elements with larger values are concentrated within the first *L* samples, and the latter *N–L* elements have non-zero values but small values. Additionally, *h* can be approximated as sparse to provide the feasibility of applying a channel estimation method based on CS theory.

Assuming the number of subcarriers in each OFDM symbol used for transmitting pilots as $N_{p}\left(N_{p}<N\right)$, the position number of the pilots is $\kappa =\left(k_{1},\cdots ,k_{N_{p}}\right)$ ($1\leq k_{1}\leq \cdots \leq k_{N_{p}}\leq N$), and the remaining $N-N_{p}$ subcarriers are applied to transmit a data signal. The set of pilot position numbers κ is called the pilot pattern, and the transmitted and received pilots are expressed as follows [23]:

(7) $$Y_{N_{p}}=X_{N_{p}}F_{N_{p}}h+W_{N_{p}}$$

where $X_{N_{p}}=diag\left[X\left(k_{1}\right),X\left(k_{2}\right)\cdots X\left(k_{N_{p}}\right)\right]$ is the transmitter pilot matrix of $N_{p}\times N_{p}$, and $F_{N_{p}}$ is the partial Fourier transform matrix of $N_{p}\times N$ extracting rows numbered $\left(k_{1},k_{2}\cdots k_{N_{P}}\right)$ from the standard Fourier transform matrix of $N_{}\times N_{}$, which is stated as follows:

(8) $$F_{N_{p}}=\frac{1}{\sqrt{N}}\left[\begin{array}{ccc} f^{k_{1}1} & \cdots & f^{k_{1}N}\\ \vdots & \ddots & \vdots \\ f^{k_{N_{p}}1} & \cdots & f^{k_{N_{p}}N} \end{array}\right]$$

where $f=e^{-j2\pi /N}$, $W_{N_{p}}$ is the Gaussian white noise vector in the frequency domain of $N_{p}\times 1$, and $Y_{N_{p}}$ is the received pilot vector of $N_{p}\times 1$. The matrix product is stated in Equation (7) as follows:

(9) $$A_{N_{p}}≜X_{N_{p}}F_{N_{p}}=\left[\begin{array}{ccc} X(k_{1})f^{k_{1}1} & \cdots & X(k_{1})f^{k_{1}N}\\ \vdots & \ddots & \vdots \\ X(k_{N_{p}})f^{k_{N_{p}}1} & \cdots & X(k_{N_{p}})f^{k_{N_{p}}N} \end{array}\right]$$

Equation (7) is then expressed as follows:

(10) $$Y_{N_{p}}=A_{N_{p}}h+W_{N_{p}}$$

Based on CS theory, the matrix $A_{N_{p}}$ of $N_{p}\times N$ can be considered an observation matrix, which is essentially a partial Fourier transform matrix formed via a weighted transmitted pilot matrix $X_{N_{p}}$. CS-based channel estimation can be assumed as the process of obtaining the received pilot vector $Y_{N_{p}}$ after a compressive measurement of *h* with $N_{p}$ pilots and then reconstructing *h* with $Y_{N_{p}}$.

#### 2.1.2. SC-FDE System Based on CS Channel Estimation

**Figure 1:**
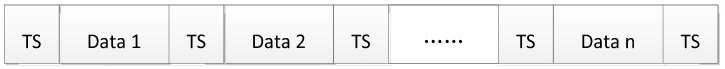
SC-FDE frame structure based on the training sequence.

A training sequence (TS) can be applied as both a guard interval and a reference signal. Figure 1 shows the frame structure of the TS-based SC-FDE system.

Generally, the training sequences of SC-FDE systems apply the same unique word (UW), and the most common form of the UW is a CAZAC sequence with a constant envelope characteristic. In order to avoid the interference of data blocks due to the multipath delay spread, reference [24] applied a dual UW (DUW) as a guard interval, which effectively improved the channel estimation performance, but the channel overhead was high because the length of each UW was equivalent to the maximum multipath delay. To decrease the channel overhead, reference [25] developed an iterative method whose total UW length was equivalent to the maximum multipath delay, but it was not suitable for fast time-varying channels due to their slow convergence, which made it impossible to acquire the channel state quickly. Reference [19] explored CS-based TDS-OFDM transmission methods. The frame structure of the TDS-OFDM system was very similar to that of SC-FDE, which utilized a known TS in the time domain as a guard interval for data blocks. The research results showed that, when the SC-FDE system applied the CS-based channel estimation method, it only needed to insert a single UW to obtain better performance than conventional methods, which improved the reliability and effectiveness of the system at the same time.

**Figure 2:**
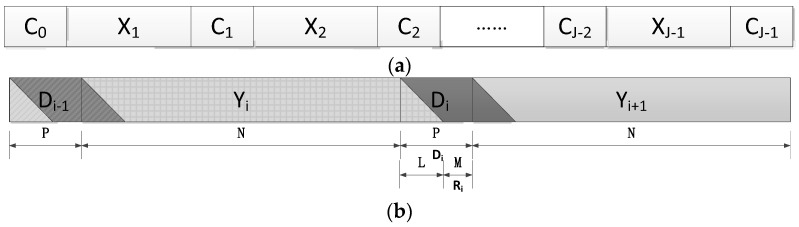
Block transmission and multipath interference. (**a**) Block transmission of transmitter signals; (**b**) multipath interference.

Figure 2a shows the block transmission structure of the TS-based SC-FDE system, where the known sequence Ci=[ci,0,c,i,1⋯,ci,P−1]T is a UW with length *P*. Therefore, for any *i* and *j*, $c_{i}=c_{j}$. Also, Xi=[xi,0,x,i,1⋯,xi,N−1]T is a payload data block with length *N*. The discrete time channel impulse response with the maximum multipath spread length *L* is assumed to be Hi=[hi,0,h,i,1⋯,hi,L]T, which satisfies *P* ≥ *L*. *M* = *P* − *L* is denoted. Figure 2b illustrates the received signal after channel transmission and time–frequency synchronization.

The received signal Di=[di,0,d,i,1⋯,di,P−1]T corresponding to a known sequence is stated as follows:

(11) $$D_{i}=\Psi_{i}H_{i}+N_{i}$$

where *N_i_* is additive Gaussian white noise, and $\Psi_{i}$ is as follows:

(12) $$\Psi_{i}={\left[\begin{array}{ccccc} c_{i,0} & x_{i,N-1} & x_{i,N-2} & \cdots & x_{i,N-L}\\ c_{i,1} & c_{i,0} & x_{i,N-1} & \cdots & x_{i,N-L+1}\\ c_{i,2} & c_{i,1} & c_{i,0} & \cdots & x_{i,N-L+2}\\ \vdots & \vdots & \vdots & \ddots & \vdots \\ c_{i,L} & c_{i,L-1} & c_{i,L-2} & \cdots & c_{i,0}\\ \vdots & \vdots & \vdots & \ddots & \vdots \\ c_{i,P-1} & c_{i,P-2} & c_{i,P-3} & \cdots & c_{i,P-L-1} \end{array}\right]}_{P\times (L+1)}$$

It is evident in matrix $\Psi_{i}$ that the last *M* samples of the received signal *D_i_* are not interfered with unknown data *X_i_*, which is known as the inter-block-interference (IBI)-free region of TS and is recorded as Ri=[di,L,d,i,L+1⋯,di,P−1]T. Also, *V_i_* is the vector that contains the last *M* samples of *N_i_*; then:

(13) $$R_{i}=\Phi_{i}H_{i}+V_{i}$$

where $\Phi_{i}$ is a Toeplitz array, stated as follows:

(14) $$\Phi_{i}={\left[\begin{array}{ccccc} c_{i,L} & c_{i,L-1} & c_{i,L-2} & \cdots & c_{i,0}\\ c_{i,L+1} & c_{i,L} & c_{i,L-1} & \cdots & c_{i,1}\\ \vdots & \vdots & \vdots & \ddots & \vdots \\ c_{i,P-1} & c_{i,P-2} & c_{i,P-3} & \cdots & c_{i,P-L-1} \end{array}\right]}_{M\times (L+1)}$$

For the SC-FDE of DUW, *M* = *L* + 1, the matrix $\Phi_{i}$ is the column full rank. Then, Equation (13) is applied for conventional methods, such as the least square (LS) and minimum mean square error (MMSE) algorithms, to estimate channel state *H_i_*. For wireless broadband channels, the non-zero elements of *H_i_* are mainly concentrated in a few positions, which are approximated as follows:

(15) $$H_{i}\left(n\right)=\sum_{k=0}^{K-1}\alpha_{i,k}\delta \left(n-\tau_{i,k}\right)\;n\in \left[0,L\right]$$

The path delays are assumed to be integers in this research. The *k*th path gain is $\alpha_{i,K}$, and the delay is $\tau_{i,K}$. As shown in Equation (15), the element value of *H_i_* is $\alpha_{i,K}$ at $\tau_{i,K}$, the value of the other elements is zero, and there are *K* non-zero elements. The path delay set is recorded as Ωi=[τi,0,τ,i,1⋯,τi,K−1], and the path gain is set as Ai=[αi,0,α,i,1⋯,αi,K−1]. Without a loss of generality, $0\leq \tau_{i,0}\leq \tau_{i,1}\leq \cdots \leq \tau_{i,k-1}\leq L-1$ can be supposed. In general, we have that *K* is much less than *L*, denoted by *K << L*; that is, the channel has a significant sparse characteristic. Therefore, channel estimation can be performed using CS methods. In CS algorithms, *R_i_* is considered the observation vector, $\Phi_{i}$ is the observation matrix, *M* is the number of observations, and *K* is the sparsity of *H_i_*. It has been proven that $\Phi_{i}$ satisfies the RIP condition required in CS theory [26].

The idea of CS is to turn the problem of solving *L* unknowns *H_i_* into the problem of estimating the 2*K* unknowns Ω*_i_* and *A_i_*, the key of which is the correct estimation of Ω*_i_*. When *M < L* + 1, the problem of solving *H_i_* in Equation (13) turns into an underdetermined equation solution problem. Since *H_i_* is a sparse variable, according to CS theory, the solution of this underdetermined equation can be obtained through the optimization problem below to obtain the estimation of channel parameter *H_i_*:

(16) $${\hat{H}}_{i}=\underset{H_{i}}{\arg\min}{\left\|H_{i}\right\|}_{0}\;s.t.\;{\left\|R_{i}-\Phi_{i}H_{i}\right\|}_{{}_{2}}^{}\leq \varepsilon$$

where ε is a noise-related non-negative constant. In recent years, the development of CS theory has provided corresponding algorithms for solving the above problem, which have mainly included relaxed convex optimization algorithms, basis pursuit (BP) algorithms, matching pursuit (MP) algorithms based on a greedy search, etc.

### 2.2. Improvement of the OMP Algorithm

#### 2.2.1. CRLB for Parameterized Channel Estimation

According to signal detection and estimation theory, for parameter estimation problems, the CRLB establishes a lower bound for the variance of any unbiased estimator; i.e., it is impossible to obtain an unbiased estimator with an estimated mean square error (MSE) smaller than the CRLB. Therefore, the difference between the MSE of the estimate and the CRLB is usually taken as an important indicator for evaluating the performance of an estimation method in both frequency offset and channel estimation [27].

When the set Ω of the channel path delay and sparsity *K* of the channel are obtained, the signal transmission model is as follows:

(17) $$R=\Phi_{\Omega }H_{K}+V$$

where *R* is the received TS of the IBI-free region with length *M,* and $\Phi_{\Omega }$ is the observation matrix of M×K, which is a sub-matrix of Φ whose column indices are elements in Ω. Also, Gaussian white noise $V=\left[v_{1},v_{2},\cdots v_{M}\right]$ obeys the distribution of $N\left(0,\sigma^{2}I_{M}\right)$, and $H_{K}$ is the column vector of K×1, giving only the channel sparse location coefficients.

Assuming $H_{K}$, the conditional probability density function (PDF) of *R* is denoted as follows [20]:

(18) $$p_{R|H_{K}}\left(R;H_{K}\right)=\frac{1}{{\left(2\pi \sigma^{2}\right)}^{M/2}}\exp\left\{-\frac{1}{2\sigma^{2}}{\left\|R-\Phi_{\Omega }H_{K}\right\|}_{2}\right\}$$

Using vector estimation theory [27], the CRLB of unbiased estimation ${\hat{H}}_{K}=\Phi_{\Omega }^{†}R$ can be introduced as expressed in Equation (19):

(19) $$CRLB=E\left\{{\left\|{\hat{H}}_{K}-H_{K}\right\|}_{2}^{2}\right\}$$

According to Equation (17), the estimated value of $H_{K}$ is obtained to satisfy

(20) $$\begin{array}{l} {\hat{H}}_{K}=\Phi_{\Omega }{}^{†}R=\Phi_{\Omega }{}^{†}(\Phi_{\Omega }H_{K}+V)\\ =\Phi_{\Omega }{}^{†}\Phi_{\Omega }H_{K}+\Phi_{\Omega }{}^{†}V=H_{K}+\Phi_{\Omega }{}^{†}V \end{array}$$

that is,

(21) $${\hat{H}}_{K}-H_{K}=\Phi_{\Omega }{}^{†}V$$

thus,

(22) $$CRLB=\left\{{\left\|{\hat{H}}_{K}-H_{K}\right\|}_{2}^{2}\right\}=E\left\{{\left\|\Phi_{\Omega }{}^{†}V\right\|}_{2}^{2}\right\}=E\left\{{\left\|{\left(\Phi_{\Omega }{}^{H}\Phi_{\Omega }\right)}^{-1}\Phi_{\Omega }{}^{H}V\right\|}_{2}^{2}\right\}$$

Considering $G=\Phi_{\Omega }{}^{H}\Phi_{\Omega }$, when all elements in $\Phi_{\Omega }$ are identically distributed random numbers with modulus 1, the diagonal elements of Gram matrix *G* are the constant *M*, and the remaining elements are normally distributed random numbers with a mean of 0 and variance of 1/*M*. Expanding the matrix operations further yields

(23) $$CRLB=E\left\{{\left\|G^{-1}\Phi_{\Omega }{}^{H}V\right\|}_{2}^{2}\right\}=K{\left(\frac{1}{M}\right)}^{2}\sigma^{2}M=\frac{K}{M}\sigma^{2}$$

From Equation (23), it can be seen that an increasing *M* decreases the CRLB; i.e., increasing the number of observations improves the probability that the OMP algorithm correctly recovers the original sparse signal. The relationship between *M* and the CRLB verified that increasing the number of observations effectively improves the performance of channel estimation. When channel multipath delay is calculated using the CS-based reconstruction algorithms, an accurate channel length can be obtained, which is usually shorter than the default maximum length; therefore, a larger number *M* of observations can be obtained, and a new observation matrix can be generated.

#### 2.2.2. Improvement of the OMP Algorithm

Knowing that the observation matrix of M×N is Φ, that the observation vector is *y,* and that the sparsity is *K*, the steps to solve the sparse signal $\hat{s}$ using the OMP algorithm are as follows:

1. Initialization parameters: residual $r_{0}=y$, index set $\Lambda_{0}=\emptyset$, the set of selected column vectors in the observation matrix $A_{0}=\emptyset$, and a number of iterations of *t* = 0.
2. *t* = *t* + 1, a column $\alpha_{j}$ of the observation matrix Φ, is searched for the elements that best match the residual according to the principle of maximum correlation, and the index set $\lambda_{t}$ is updated, which satisfies the following:
  (24) $$\lambda_{t}=\arg\underset{j=1,2,3\ldots N}{\max}\left|\langle r_{t-1},\alpha_{j}\rangle \right|$$
  where $\alpha_{j}$ denotes the *j*th column of observation matrix.
3. The index set $\Lambda_{t}=\Lambda_{t-1}\cup \;\;\;\left\{\lambda_{t}\right\}$ is updated, and $A_{t}=A_{t-1}\cup \alpha_{\lambda_{t}}$; where *t* denotes the current number of iterations and the number of elements in the current index set.
4. The least square solution is found:
  (25) $${\hat{s}}_{t}=\underset{s}{\arg\;\;\min}\left\|y-A_{t}s_{t}\right\|={\left(A_{t}^{H}A_{t}^{}\right)}^{-1}A_{t}^{H}y$$
5. New approximations and residuals are calculated: $r_{t}=y-A_{t}{\hat{s}}_{t}$.
6. If t<K, the process returns to step 2; otherwise, iteration is stopped and returns to step 7.
7. The non-zero element position set of $\hat{s}$ obtained from the reconstruction is $\Lambda_{t}$, and the value of the non-zero element is the solution of the last iteration ${\hat{s}}_{t}$.

The OMP algorithm guarantees the optimality of each iteration, with the number of iterations equal to the sparsity value. The main ideas of the SOMP and OMP algorithms are the same, and their difference lies only in step 2. When searching for the column index of the observation matrix that most matches the residual, corresponding to a number of joint sparse signals, the inner products of the residual and the column are summed, and then the column corresponding to the maximum value is selected. The other steps are exactly the same. All solutions of sparse signals are obtained via the LS rule.

It should be noted that, when the OMP algorithm is applied to reconstruct the original signal, the observation matrix Φ is known. According to Equation (14), $\Phi_{i}$ is the matrix of M×(L+1), where *L* is channel length, which is related to the maximum delay spread of the channel. However, in practical applications, *L* is unknown and can only be obtained by estimating the realistic maximum delay of the channel, which is usually greater than the actual channel length. Reference [22] proposed a PIA-OMP algorithm based on a priori information, which improved OMP reconstruction accuracy and, thus, channel estimation performance. By inserting the cyclic prefix of the first TS as a guard Interval in the header of a transmitted frame, this method obtains a priori information such as the channel length and sparsity, but it sacrifices some spectral efficiency. In addition, it assumes a constant CIR within a frame, which has limitations for application in fast-fading channels.

In this research, we propose an improved scheme that can be applied to both the OMP and SOMP algorithms. It can significantly improve the performance of the reconstruction algorithm without sacrificing spectral efficiency. Based on the maximum possible delay, the channel length is preset to be *L,* and a more precise channel length is calculated when the path delay set is obtained through several iterations. The set is estimated to be ${\hat{\Omega }}_{i}=\left\{{\hat{\tau }}_{0},{\hat{\tau }}_{1},\cdots {\hat{\tau }}_{K-1}\right\}$; then, the channel length is $\hat{L}={\hat{\tau }}_{K-1}$ and $\hat{L}\leq L$. Therefore, the length of the IBI-free region *M* is enhanced, and thus, the observation matrix is updated as follows:

(26) $${\Phi^{\prime }}_{i}={\left[\begin{array}{ccccc} c_{i,\hat{L}} & c_{i,\hat{L}-1} & c_{i,\hat{L}-2} & \cdots & c_{i,0}\\ c_{i,\hat{L}+1} & c_{i,\hat{L}} & c_{i,\hat{L}-1} & \cdots & c_{i,1}\\ \vdots & \vdots & \vdots & \ddots & \vdots \\ c_{i,P-1} & c_{i,P-2} & c_{i,P-3} & \cdots & c_{i,P-L-1} \end{array}\right]}_{M^{\prime }\times (\hat{L}+1)}$$

where $M^{\prime }=P-\hat{L},\mathrm{and}\;M^{\prime }>M$. The number of rows of ${\Phi^{\prime }}_{i}$ is increased, and the number of columns is decreased compared to the initialized observation matrix given in Equation (14). Substituting Equation (26) into Equation (13) yields the following:

(27) $$R_{i}{}^{\prime }=\Phi_{i}^{\prime }H_{i}^{\prime }+V_{i}^{\prime }$$

where $R_{i}{}^{\prime }$ is the IBI-free region signal with length $M^{\prime }$, which is the observation vector in the OMP algorithm and is recorded as R′i=[di,L^,d,i,L^+1⋯,di,P−1]T,H′i=[hi,0,h,i,1⋯,hi,L^]T, and ${V^{\prime }}_{i}$ is the vector that contains the last $M^{\prime }$ samples of the additive Gaussian white noise signal *N_i_*. According to Equation (23), and due to the increase of *M*, the CRLB is decreased, and according to Equation (27), the recalculation of the channel path gain based on the LS criterion provides a certain performance gain. Based on the new vector $R_{i}{}^{\prime }$ and the observation matrix ${\Phi^{\prime }}_{i}$, as well as the estimated path delay set ${\hat{\Omega }}_{i}$, the overdetermined equation ${R^{\prime }}_{i}={{\Phi^{\prime }}_{i}|}_{{\hat{\Omega }}_{i}}{H^{\prime }}_{i}$ is solved, where ${{\Phi^{\prime }}_{i}|}_{{\hat{\Omega }}_{i}}$ is the column set of ${\Phi^{\prime }}_{i}$ with the column index in the set ${\hat{\Omega }}_{i}$, which can be obtained according to the least squares criterion:

(28) $${\hat{H}}_{i}={\left({{\Phi^{\prime }}_{i}|}_{{\hat{\Omega }}_{i}}{}^{H}{{\Phi^{\prime }}_{i}|}_{{\hat{\Omega }}_{i}}\right)}^{-1}{{\Phi^{\prime }}_{i}|}_{{\hat{\Omega }}_{i}}{}^{H}{R^{\prime }}_{i}$$

**Figure 3:**
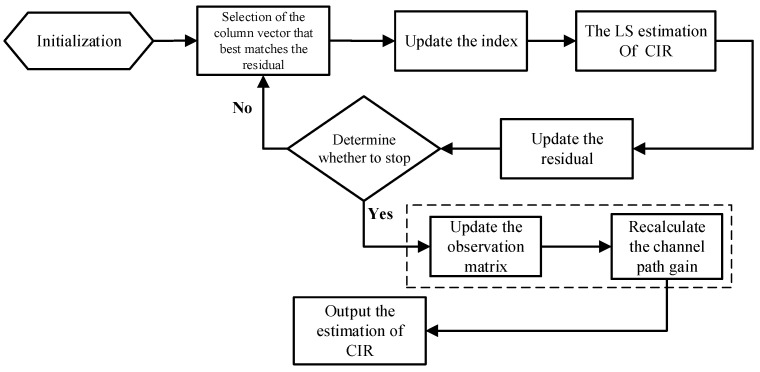
The process of the improved OMP algorithm.

In summary, Figure 3 shows the process of the proposed scheme for the OMP and SOMP algorithms. The modules of the conventional OMP and SOMP algorithms are outside the dashed box, and the improvement parts are inside it. Compared with conventional algorithms, better estimation performance is obtained by employing the proposed scheme. Since both methods fully utilize the IBI-free region signal as an observation vector, the proposed method in this research is able to achieve the same performance gains as the method in reference [22], but it does not require additional pilots. Furthermore, since matching pursuit algorithms are all based on obtaining accurate path delay estimates before calculating the path gain, the proposed method is also applicable to other matching pursuit algorithms.

Algorithm 1 summarizes the steps of the proposed improved OMP algorithm. The initial observation matrix Φ is $\Phi_{i}$ in Equation (14) with a size of M×(L+1), the observation vector *y* is *R_i_* in Equation (13), the sparse signal *s* is *H_i_*, the sparsity level is *K*, and the updated observation matrix is ${\Phi^{\prime }}_{i}$ in Equation (26).
**Algorithm 1:** Proposed improved OMP algorithm.1. $\mathrm{Initialization}\;\mathrm{parameters}:\;\mathrm{residual}\;r_{0}=y$$,\;\mathrm{index}\;\mathrm{set}\;\Lambda_{0}=\emptyset$$,\;\mathrm{the}\;\mathrm{set}\;\mathrm{of}\;\mathrm{selected}\;\mathrm{column}\;\mathrm{vectors}\;\mathrm{in}\;\mathrm{the}\;\mathrm{observation}\;\mathrm{matrix}\;A_{0}=\emptyset$, and number of iterations *t* = 0. 2. *t* = *t* + 1, $a\;\mathrm{column}\;\alpha_{j}\;\mathrm{of}\;\mathrm{the}\;\mathrm{observation}\;\mathrm{matrix}\;\Phi ,\;\mathrm{is}\;\mathrm{searched}\;\mathrm{for}\;\mathrm{the}\;\mathrm{elements}\;\mathrm{that}\;\mathrm{best}\;\mathrm{match}\;\mathrm{the}\;\mathrm{residual}\;\mathrm{according}\;\mathrm{to}\;\mathrm{the}\;\mathrm{principle}\;\mathrm{of}\;\mathrm{maximum}\;\mathrm{correlation},\;\mathrm{and}\;\mathrm{the}\;\mathrm{index}\;\mathrm{set}\;\lambda_{t}$ is updated, which satisfies the following:     $\lambda_{t}=\arg\underset{j=1,2,3\ldots N}{\max}\left|\langle r_{t-1},\alpha_{j}\rangle \right|$ 3. The index set $\Lambda_{t}=\Lambda_{t-1}\cup \;\;\;\left\{\lambda_{t}\right\}$ and the set of selected column vectors $A_{t}=A_{t-1}\cup \alpha_{\lambda_{t}}$ are updated, where *t* denotes the current number of iterations and the number of elements in the current index set. 4. The least square solution is found:     $\hat{s_{t}}=\underset{s}{\arg\;\;\min}\left\|y-A_{t}s_{t}\right\|={\left(A_{t}^{H}A_{t}^{}\right)}^{-1}A_{t}^{H}y$ 5. $\mathrm{New}\;\mathrm{approximations}\;\mathrm{and}\;\mathrm{residuals}\;\mathrm{are}\;\mathrm{calculated}:\;r_{t}=y-A_{t}{\hat{s}}_{t}$. 6. If t<K, the process returns to step 2; otherwise, iteration is stopped and returns to step 7. 7. $\mathrm{The}\;\mathrm{non}-\mathrm{zero}\;\mathrm{element}\;\mathrm{position}\;\mathrm{set}\;\mathrm{of}\;\hat{s}\;\mathrm{obtained}\;\mathrm{from}\;\mathrm{the}\;\mathrm{reconstruction}\;\mathrm{is}\;\Lambda_{K}=\left\{\lambda_{0},\lambda_{1},\cdots \lambda_{K-1}\right\}$$,\;\mathrm{denotes}\;\hat{L}=\max\left(\Lambda_{K}\right)$$,\;\mathrm{and}\;\mathrm{the}\;\mathrm{observation}\;\mathrm{matrix}\;\Phi ={\Phi^{\prime }}_{i}$ and observation vector $y={R^{\prime }}_{i}$ are updated. 8. The set of column vectors of Φ is denoted as ${\Phi |}_{\Lambda_{K}}$; with columns indexed using elements in $\Lambda_{K}$, the least square solution is found:     $\hat{s}={\left({\Phi |}_{\Lambda_{K}}{}^{H}{\Phi |}_{\Lambda_{K}}\right)}^{-1}{\Phi |}_{\Lambda_{K}}{}^{H}y$

It should be noted that, when applying CS-based channel estimation to OFDM systems, a mathematical model is established, as stated in Equation (10). When applying a greedy pursuit algorithm to reconstruct the time domain CIR, the observation vector length is equal to the number of frequency pilots, so all reference signals are utilized, and there is no possibility of further increasing the observation number. Therefore, the improved method is not applicable to OFDM systems using frequency domain pilots as reference signals. In particular, since the TDS-OFDM system uses the time domain TS as reference signals for synchronization and channel estimation, it is similar in structure to the SC-FDE system, and thus the improved scheme is also applicable to the TDS-OFDM system based on CS channel estimation.

## 3. Results and Discussion

This research sets up an SC-FDE system simulation platform to simulate the proposed improved algorithm. Multipath channel models are selected from tapped delay models, and the channel parameters of four paths and six paths are set according to the ionospheric channel characteristics of shortwave communication and the typical urban area channel model of wireless communication COST207 [28], as summarized in Table 1, where channel sparsity is the number of multipaths. Assuming that the system is ideally synchronized, the main parameters of the transmitter are shown in Table 2.

Channel length and sparsity are selected according to the following considerations. Based on the properties of ionospheric channels [29], the maximum multipath delay spread is $\tau_{\max}=5\;\mathrm{ms}$. Considering that the system sampling rate is $f_{s}=8\;\mathrm{KSps}$, the tap number of the tapped delay channel model, and also the initialized value of the channel length, is $L=f_{s}\times \tau_{\max}=40$. ITU-R suggests test channels for the simulation of the performance of the shortwave digital communication system, and ITU-R F.1487 defines ten test channels at different latitudes and under different environmental conditions. These test channels are all Watterson models with a two-path tapped delay [29]. Six channel models are suggested according to the DRM digital broadcasting standard [30], in which the typical shortwave channel model is a four-path channel, and sparsity of *K* = 4 is chosen to better verify the reconstruction performance of the CS algorithm. Similarly, according to the typical urban area of the COST207 model, the maximum multipath delay spread is $\tau_{\max}=5\;\mu s$, and the system sampling rate is taken as $f_{s}=10\;\mathrm{MSps}$, from which the initialized value of channel length is $L=f_{s}\times \tau_{\max}=50$. The typical urban area channel model given via COST207 usually has six significant paths; therefore, the sparsity is chosen to be *K* = 6. The iterations of the OMP and SOMP algorithms are equal to the sparsity level, and our simulations assume that the channel sparsity is known.

In order to illustrate the superiority of the proposed scheme, the improved OMP and SOMP algorithms (with jointness *J* = 16) were simulated and compared with the MMSE method [24] and conventional OMP [9] and SOMP [16] algorithms in terms of both spectral efficiency and channel estimation performance, respectively. When the MMSE method is applied, TS is a dual UW, which is a Chu sequence with a constant envelope property [31]. The first is used to resist multipath interference due to unknown data, and the second is applied as the reference signal for channel estimation. When the CS-based methods are used, UW is a pseudo-random (PN) sequence. The UW length needs to be longer than the channel maximum delay spread, and it is set to *P* = 64 and *P* = 74 for two channel conditions, respectively.

**Figure 4:**
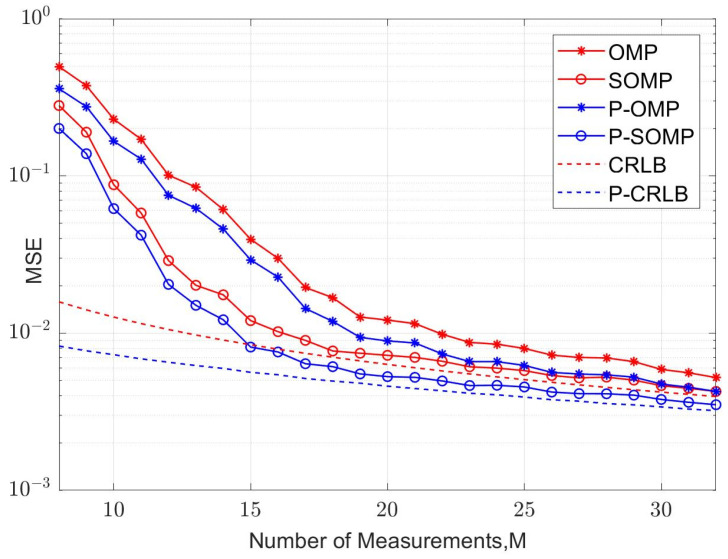
MSE corresponding to different observation numbers (K = 4, SNR = 15 dB).

**Figure 5:**
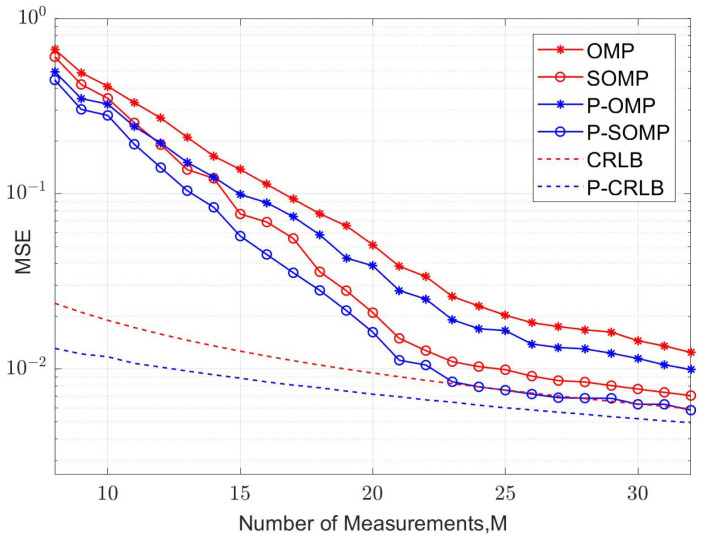
MSE corresponding to different observation numbers (K = 6, SNR = 15 dB).

In our simulation, fifteen symbol blocks were used for each simulation run, and each symbol block consisted of a TS of *P* samples and a data block of *N* samples. The average values of the MSE and bit error rate (BER) were calculated using 5000 simulation runs. The curves of the MSE versus the number of observations for conventional and improved algorithms are illustrated in Figure 4 and Figure 5 for four-path and six-path channel conditions, respectively, with a fixed signal-to-noise ratio (SNR). The SNR was chosen to be 15 dB. P-OMP and P-SOMP in the legend represent the improved algorithms proposed in this research. In the figure, the red and blue dashed lines are the CRLB using conventional and improved methods, respectively. Obviously, the channel estimator using improved algorithms reduces the CRLB and obtains a smaller MSE, which effectively improves the estimation performance.

As shown in Figure 4, the OMP algorithm achieves a mean square error of $MSE=10^{-2}$ when *M* = 22 for the four-path channel model. However, the P-OMP algorithm achieves a mean square error of $MSE=10^{-2}$ when *M* = 19. Similarly, the numbers of observations required for the SOMP and P-SOMP algorithms to achieve a mean square error of $MSE=10^{-2}$ are *M* = 17 and *M* = 15, respectively. In other words, the improved methods achieve the same performance as the conventional methods using fewer observations; however, at a constant observation number, the improved methods can significantly decrease the MSE and improve the performance of the channel estimator.

It is also evident that the MSEs of the SOMP methods applied to recover the joint sparse signal are closer to the CRLB than the OMP methods; that is, SOMP is able to utilize the temporal correlation of the channel state information (CSI) in consecutive frames, and it can use a shorter observation vector to achieve the same reconstruction probability as the OMP algorithm. When the observation vectors are the same as those of the OMP algorithms, the SOMP methods effectively reduce the MSEs and obtain more superior reconstruction performance for a sparse signal.

**Figure 6:**
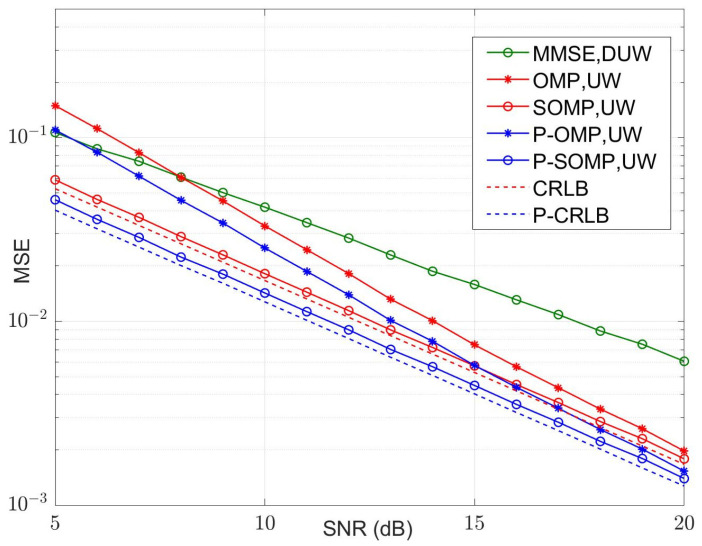
MSE with different SNR values (K = 4, M = 24).

**Figure 7:**
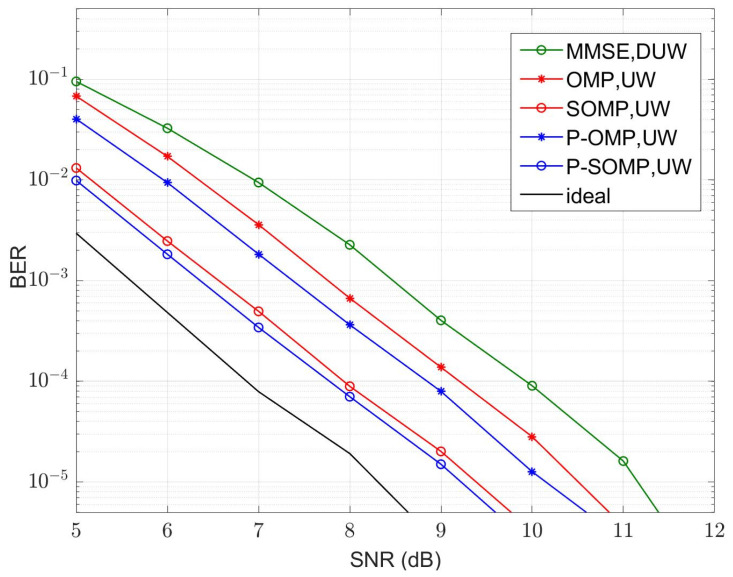
BER with different SNR values (K = 4, M = 24).

**Figure 8:**
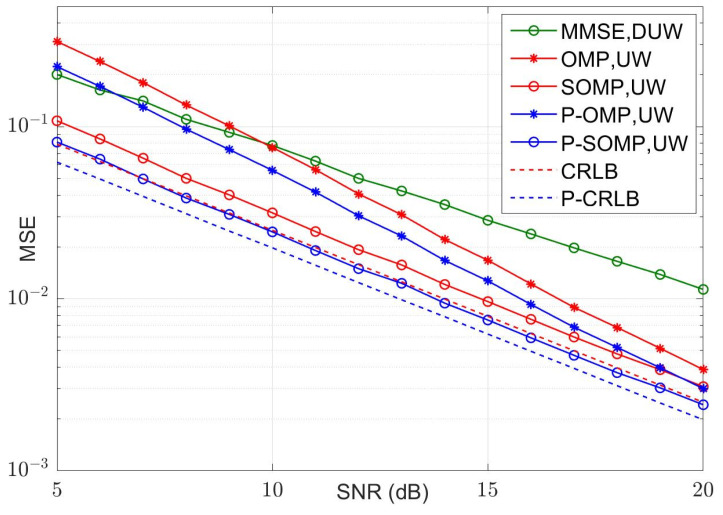
MSE with different SNR values (K = 6, M = 24).

**Figure 9:**
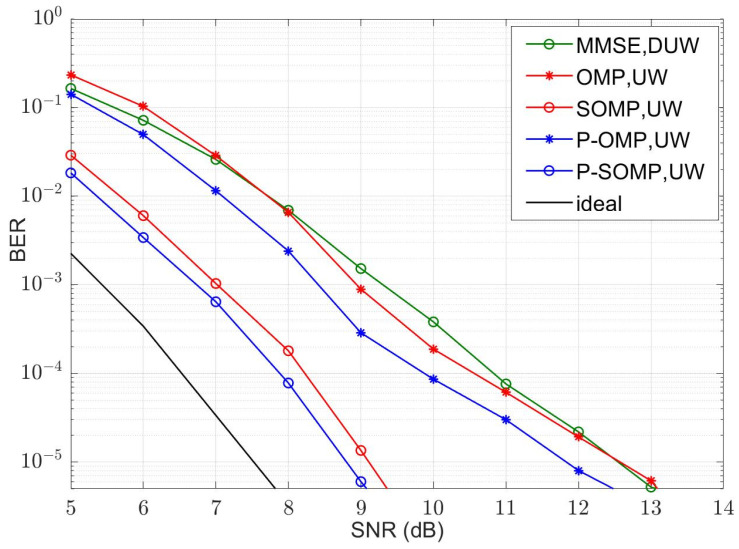
BER with different SNR values (K = 6, M = 24).

Figure 6, Figure 7, Figure 8 and Figure 9 illustrate the curves of the MSE and BER of different estimation methods with respect to the SNR, according to the two channel conditions. As shown in Figure 6 and Figure 8, the MSEs of the CS-based methods are lower than those of an MMSE method, except that the conventional OMP algorithm is not as good as MMSE at using a dual UW at a low SNR. Compared with the conventional OMP and SOMP algorithms, the improved algorithms pull down the CRLB and, thus, achieve lower MSEs. For the four-path channel, as illustrated in Figure 6, at $MSE=10^{-2}$, P-OMP achieves a SNR gain of about 1.0 dB compared to OMP and P-SOMP achieves a SNR gain of about 1.1 dB compared to SOMP. As shown in Figure 7, using the conventional OMP and SOMP algorithms, SNR gains of about 0.7 dB and 1.8 dB are achieved at $BER=10^{-5}$ compared to the MMSE, respectively. When adopting the improved algorithms, an extra 0.3 dB and 0.2 dB in SNR gains can be obtained compared to the conventional algorithms, respectively. For the six-path channel, Figure 8 illustrates that, at $MSE=10^{-2}$, P-OMP achieves a SNR gain of about 0.9 dB compared to OMP, and P-SOMP achieves a SNR gain of nearly 1.1 dB compared to SOMP. In Figure 9, it is seen that the BER performance using the conventional OMP algorithm is essentially the same as that using MMSE. However, compared with MMSE, conventional SOMP achieves a SNR gain of about 3.4 dB at $BER=10^{-5}$. Compared to conventional methods, additional SNR gains of about 0.8 dB and 0.3 dB, respectively, are obtained via the improved methods.

In terms of algorithm complexity, MMSE estimation requires a matrix-inverse operation with a time complexity of O(*P*^3^), and the complexity is higher for larger matrix orders. The application of iterative methods such as most rapid descent and conjugate gradient methods are able to avoid the matrix inverse, and channel estimation is realized by solving system equations. It was found that those methods converge faster, with O(*P*^2^) computations per iteration. For CS-based schemes, the main part of both conventional and improved algorithms lies in the iteration of a greedy algorithm. The iteration numbers of both algorithms are the same; they are equal to the channel sparsity *K*. The time complexities of the conventional OMP and SOMP algorithms are both O(*KML*), and compared with conventional methods, the improved methods only add the step of solving for the exact path gain according to the maximum likelihood rule (MLR), as stated in Equation (28). The increased time complexity is O(*M*^′^*K*^2^ + *K*^3^) [19]; i.e., the computational time is exponentially increased with the increase in sparsity. For wireless channels with significant sparsity, we have *K* << *L*, and the computations achieved via the improved algorithms are acceptable. The observation vector length *M*^′^ for calculating the path gain has a smaller effect on the complexity. The time complexities of different estimation schemes are summarized in Table 3, where the complexity of MMSE is based on the computation of a standard matrix inversion method.

Table 4 summarizes the length and overhead of the reference signals for different estimation methods. Compared with MMSE, which uses dual UWs as reference signals, CS-based methods can reconstruct channel state information with a high probability using a single UW as a reference signal while decreasing the reference signal overhead and greatly improving the spectrum efficiency, so they are very conducive to the effective transmission of wireless communication systems.

In summary, when using CS-based methods, compared with the dual UW-based MMSE method, the BER performance of the channel estimator is effectively improved with a substantial reduction of the reference signal; i.e., the effectiveness and reliability of the communication system are simultaneously enhanced. Compared with the conventional OMP and SOMP algorithms, the proposed scheme pulls down the CRLB and further improves the performance of channel estimation. Therefore, adopting CS-based estimation methods and applying an improved algorithm are of great practical significance for the reliable transmission of numerous wireless channels with sparse characteristics.

## 4. Conclusions

The OMP algorithm is an important greedy algorithm that is more extensively applied to CS-based channel estimation. This research model’s wireless channel estimation problem, as a sparse signal reconstruction problem, analyzes the factors affecting OMP algorithm performance, proposes an improved scheme applicable to SC-FDE systems, which is applied to the OMP and SOMP algorithms, and finally develops system simulations and experiments for the two channel models. The simulation results show that, compared with the conventional MMSE estimation method, CS-based methods significantly save on the reference signal overhead and improve channel estimation performance. Therefore, they improve the effectiveness and reliability of the communication system at the same time. Also, compared with the conventional OMP and SOMP algorithms, the improved algorithms use longer observation vectors to calculate the path gain after obtaining the accurate path delay, which lowers the CRLB, and they are able to further enhance the performance of the MSE and BER in channel estimation, so they further improve the reliability of the communication system effectively.

The limitation of the proposed scheme is that it assumes the known channel sparsity, which is unavailable in most fast-varying systems. In practice, a rough estimate of the sparsity can be obtained based on the LS criterion. In addition, the improved algorithms increase the complexity of the reconstruction algorithm, and the computation time increases exponentially with the increase in sparsity, but the additional computations are acceptable for the estimation of wireless channels with significant sparsity.

The improved scheme is not applicable to OFDM systems employing frequency domain pilots, but it can be applied to TDS-OFDM systems and is applicable to the improvement of other matching pursuit algorithms. As summarized, the proposed scheme is of great significance for the reliable transmission of wireless communication systems based on a transmission block structure employing time domain pilots.

## Figures and Tables

**Table 1:** Channel model parameters.

Channel Sparsity (*K*)	4	6
Channel length (*L*)	40	50
Path delay ($\tau_{k}$)	Random	Random
$\mathrm{Path}\;\mathrm{gain}\;(\alpha_{k}$)	[0, −3, −6, −9]	[−3, 0, −2, −6, −8, −10]
$\mathrm{Doppler}\;\mathrm{shift}\;(f_{d}$)	1 Hz	100 Hz

**Table 2:** Transmitter parameters.

Channel Sparsity (*K*)	4	6
$\mathrm{Sample}\;\mathrm{rate}\;(f_{s}$) (Hz)	$8\times 10^{3}$	$10\times 10^{6}$
Modulation	QPSK	QPSK
Channel coding	Convolutional encoding	Convolutional encoding
Code rate	0.5	0.5
Training sequence	Chu/PN	Chu/PN
IBI-free region length (*M*)	24	24
Training sequence length (*P* = *M* + *L*)	64	74
Data block length (*N*)	256	256

**Table 3:** Time complexities of different channel estimation schemes.

Channel Estimation Method	Time Complexity
MMSE	O(*P*^3^)
OMP/SOMP	O(*KML*)
P-OMP/P-SOMP	O(*KML*) + O(*M*^′^*K*^2^ + *K*^3^)

**Table 4:** Reference signals and overhead for different channel estimation schemes.

Channel Estimation Method	MMSE	OMP/P-OMP		SOMP/P-SOMP (*J* = 16)	
Data block length (*N*)	256	256		256	
Reference signal	DUW	UW		UW	
Reference signal length (*P*)	128	*K* = 4	*K* = 6	*K* = 4	*K* = 6
		64	74	64	74
*P*/*N*	50%	25%	28.9%	25%	28.9%

## Data Availability

Data are contained within the article.
